# Reliance on Facebook for news and its influence on political engagement

**DOI:** 10.1371/journal.pone.0212263

**Published:** 2019-03-19

**Authors:** Clarissa C. David, Ma. Rosel S. San Pascual, Ma. Eliza S. Torres

**Affiliations:** 1 College of Mass Communication, University of the Philippines Diliman, Diliman, Quezon City, Philippines; 2 School of Languages, Humanities, and Social Sciences, Mapua University, Intramuros, Manila, Philippines; Institute for Complex Systems, CNR, ITALY

## Abstract

This paper examines the link between reliance on Facebook for news, political knowledge, and political engagement in the Philippines. We tested five hypotheses using data gathered from an online survey of 978 Filipinos conducted from February 1 to March 31, 2016. Findings support the hypothesis that those who rely less on social media as a news source exhibit higher levels of perceived knowledge about politics than those who rely more on it for news. Controlling for traditional news use, following political officials or institutions on social media is associated with higher levels of political interest and engagement, those with more politically active friends on Facebook have higher levels of exposure to political content online, and there is a positive correlation between Facebook being a source of information about politics and discussing politics more often with others. However, the hypothesis that those with more friends on their network who are politically active, will have greater political knowledge and more political engagement than those who have few politically active friends on their Facebook network is not supported.

## Introduction

High levels of engagement in politics are a sign of a healthy democracy, where the citizenry is empowered and interested in being involved in matters of the State. More than the overall level of involvement in politics, it is the equitable distribution of this participation that is important. Inequitable political participation may result in a government that supports only the interests of those who participate. (e.g., [1])

Young citizens are notoriously difficult to engage in political life, often setting aside politics for entertainment (e.g., [2–4]) and they are often thought to be less engaged than older citizens. In the new media environment however, young citizens have turned en masse to social media for news about politics and public affairs.[5]

Social media sites, the most popular of which is Facebook (FB), allow users to build networks of contacts from individual or group profiles.[6] It is a legitimate venue for engagement in civic and political life, and in the most developed countries, now an important source of political news. Social media’s role in the complex relationship between news use and political engagement has become the subject of much research, with most findings suggesting that platforms such as FB have positive influences on outcomes related to politics (e.g., [7]).

In the Philippines, a developing country with a high poverty rate, roughly 58% are Internet users and more than 90% of these are on FB.[8] What are the implications of FB use for news consumption and political participation in the context of a developing country with a traditionally low level of news use?

This paper seeks to examine the influence of reliance on FB for news on political engagement, independently of use of traditional news sources. We attempt to examine various sources of information about news and political affairs on FB, including government agency FB pages and news sites. Specifically, it hypothesizes that for individuals who consider it a news source, those who follow political officials and institutions on social media and those with more politically active social media friends in their online news network are more knowledgeable and more highly engaged, independently of their traditional news consumption.

## News online and political engagement

There is a mature literature around the question of whether Internet use is consequential to political engagement, and most studies conclude that its influence is generally positive. It has been found to increase political engagement,[9–10] increase knowledge about political campaigns,[11] and improve voting or other types of participation.[12–13] Internet-based expression of political views are linked to offline forms of participative behaviors.[14–18] Evidence is likewise accumulating that these relationships extend to the social media platforms of FB and Twitter, where users are connected as they share and engage with various kinds of content, including news and public affairs.

Our research has a specific interest in the use of FB for news consumption, and its influence on political engagement. The platform is now the main driver of traffic to news websites, and while it does not generate original news content and the company has made specific statements that they are not a news or media company, FB has grown into an important medium for news.

In a 2016 study, Pew Research Center accounted that 62% of adults in the US get news from social media.[19] Majority of the social media news consumers that they surveyed reported that they only get their news from one social media site—FB. In fact, the same study reported that 66% of FB users get news from FB. (ibid)

Some recent work on the information gaps created by the online news environment suggests that less educated and less politically inclined citizens do not benefit with as much gain in political knowledge from online formats compared to print, while the more educated citizens gain knowledge regardless of the medium.[20] In the mid-2000s, scholars were already concerned that the expansion of choice in news content and sources, through news websites, would lead to a widening of knowledge gaps (e.g., [21]). Experimental and survey studies that examine information disparities between newspaper reading and online news reading mostly find that knowledge is better retained when reading newspapers compared to reading online news websites. Online formats no longer had the traditional cues present in print, such as story importance cues.[22] Moreover, organization by topical menus meant that users were more likely to stay within their issue interests and in the end become less exposed to issues that fall outside their core set of concerns.[23]

The entry of social media as a source of news, and the growing reliance of the general public on these channels for news consumption, re-opens this line of inquiry. FB is considered as a new layer of gatekeeping, its algorithm and the preferences of a user’s friends list determine whether you see a story and how many times it will cross your feed. It remains an open question whether political news read through FB opens up a new market for this content or only provides an additional channel of access to those who are already news consumers in other media. At least for the young, those who we consider as digital natives, FB is a natural part of their media habits and for many of them, there could be a reliance on the platform for exposure to political news. It is thus as important to know the quality of their use of this media platform.[24]

This study is interested in the idea of reliance on FB for information, that is, the degree to which users are dependent on FB for news so that without it, they would have limited exposure. We hypothesize that those who are reliant on FB were not politically engaged and interested to begin with, and thus, would have little political knowledge outside of their FB feed.

The empirical association between political knowledge and interest, engagement, and participation in civic and political activities is well-established (e.g., [25–26]). Understandably, one’s decisions concerning participation depends on relevant political knowledge.[27] The positive relationship between Internet use and participation is stronger with interactive platforms like blogs and social networks rather than static platforms online.[28] Social media use, especially for political content, has been found to correlate with various forms of political participation (e.g., [29–30]), although some conflicting findings also question whether these are effects and whether they are direct or indirect.[26] Further, others contend that the Internet has caused many to retract from public life.[12] Gil de Zuniga, Jung, and Valenzuela find evidence that information seeking on social media sites are predictive of online and offline participatory behaviors.[27] These existing studies guide our further hypothesis that following various sources of political news on FB feeds, like government institutions, pages of politicians and candidates, or news pages, would be associated with higher levels of political engagement and interest, including higher levels of political discussion with others.[31–32] These associations are hypothesized to exist independently of traditional news media exposure.

Reliance on FB for political news content would mean that one’s news exposure is influenced more strongly by the mechanisms on the platform that determine what kinds of news and political content appears on a person’s news feed. The main factors that influence the content you would see on your feed may be likened to filtering: the stories that people in your social network share, the stories that are viral or trending (popular), and those that are pushed by paid advertising.[27] Each of these factors interact with the other, for instance news stories that are “liked” by people in a social network and are trending in the broader environment, are more likely to appear on your feed.

It is possible that political content exposure on social media is amplified in effect compared to traditional media. People are able to translate their interests into participation because what they see include opinions and emotional appeals of people that they know. We are more inclined to pay attention to utterances from friends and family, and more likely to think more carefully about arguments that come from them rather than strangers or even reporters. Groups now turn to social media for recruitment, to expand their constituency.[31, 33] This line of argument privileges social capital as a key enabler of participative behaviors, both online and offline.[7, 27, 34]

Since content shared and posted across FB are diverse, and only a portion of it is political in nature, we expect that those whose social networks have more politically active people will have greater exposure to news content than others, and that this would be associated with higher levels of political knowledge and engagement. This is consistent with findings of Wolfsfeld, Yarchi, and Samuel-Azranal,[35] which show that social media-based political content exposure is more highly correlated with traditional measures of political participation than regular media. The association is much stronger even, between social media-based political news exposure, and digital political participation.

## Facebook in the Philippines

The Philippines just entered its status as a “middle-income” country, from a long history of being a poor country with a large proportion of the population living in poverty (around 22%). It is a country with low levels of high school and college graduation rates and generally low access to many forms of technology. Most recent data show laptop and desktop computer ownership/access at only 24.3% of households across the country,[36] most schools do not have computers in classrooms, and most students do not have access to one. However, the rapid adoption of smartphones and the sharp decline in the costs of smartphone units that are capable of Internet connection led to an expansion of internet access in the Philippines.

Officially, Internet penetration rate in the country is less than 40% for a country of 101 million people. However, according to FB, there are over 50 million Filipinos in the country on their platform. Among those that report having access to the Internet, over 94% report having a social media account and the vast majority of them are on FB.[37] As such, the Philippines is often characterized as the social media capital of the world, on top of its reputation as the texting capital of the world. The leading telecommunication companies in the country offer access to the FB app on smartphones without charging for data usage; this further grew the population of Filipinos on FB.

The Philippines has a history of low levels of political knowledge acquired from traditional media, no doubt owing to the very low circulation rates of newspapers (at best, 15% read the paper regularly). Most news consumption happens through television, and these days, social media. Compared to FB growth even in developed countries, FB in the Philippines grew rapidly into the main driver of news traffic among the most prestigious news outlets in the country. A paper (authors) on the role of FB on news reporting in the country reveals that among the top 4 news websites, FB-referred traffic accounts for between 60%-90% of clicks into news sites. This was a year ago and it is reasonable to think that these percentages have grown since. Television remains the number 1 news source across the country; but among those with Internet access, it is likely that the majority of news consumption is FB-driven.

This unique situation of the Philippine news reading public makes it a good venue to study the potential implications of reliance on FB for political news. Fresh out of a contentious election with controversial results, FB-based campaigning and political activism have flourished and thrived, some say, to the detriment of civilized political debate. Online discussions and comments grew vicious and sometimes violent, and full of political–and personal–vitriol, thus recently prompting the Senate to open a hearing on the problem of social media’s effect on culture. Clearly FB has an important role to play in the Philippines’ news use, political knowledge, and engagement with politics, providing an interesting context for understanding the relationships hypothesized in this study.

## Hypotheses

Based on where we are now in the literature on social media, political knowledge, and engagement, this research seeks to contribute to this fast-growing area by looking specifically at FB as a news source, reliance on it as a source of news and information, and its correlation with political engagement.

This research is guided by an interest in answering the research question: Is FB use associated with political engagement, knowledge, and opinions about important political issues? To systematically investigate how these concepts relate to each other, the following hypotheses are posed:

H1: Those on social media who rely less on FB as a news source exhibit higher levels of perceived knowledge about politics than those who rely more on it for news.

Hypothesis 1 is posed in relation to a conceptualization that “reliance” means using Facebook as a source of information without the benefit of using other traditional media sources, such as television or newspapers. As reviewed above, the Philippine news market is not strong, there has historically been low newspaper reading publics. Thus, high reliance individuals are those that, in the extreme case, only get their political knowledge from Facebook while those with low reliance may still get knowledge from the platform but also get knowledge from other news sources. The direction of the hypothesis is consistent with extant literature showing higher knowledge among those with higher news media consumption [10, 11].

H2: Following political officials or institutions on FB will be associated with higher levels of political interest and engagement.

One of the ways Facebook is used by political actors and institutions, as well as media, is to create and maintain official institutional and personal accounts which are used to publicize programs, accomplishments, and information pertaining to an agency or a politician. We hypothesize that when users are following such pages on FB, they are more likely to have higher levels of engagement and interest because the active and conscious decision to click “follow” on a page indicates a willingness to regularly see information on those pages. In certain markets where survey data are available, following politicians on social media is as high as 35% in some countries, and that the reason for following is an interest in hearing directly from politicians and learning more detailed information [38].

H3: Having more politically active friends on FB will result in higher levels of exposure to political content online.

Consistent with empirical literature on political discussion and online political engagement [39], as well as what is known about the FB algorithms that prioritizes on individual newsfeeds stories shared within networks [40], we apply these ideas to predict that having more politically active friends on FB will result in higher exposure levels to political content. That is, if an FB user has a large number of friends who are politically active, interested, and engaged, those same friends will be posting and reposting about politics, news, and current affairs, which means that even if the primary user is not necessarily politically interested, s/he would have a higher likelihood of being exposed to political content by having online social networks that are politically interested.

H4: Independently of traditional news consumption, those who are exposed to news through social media will discuss politics more often with others than those who are not exposed to news through social media.

When publics on FB encounter political information on the platform, we hypothesize that they are more likely to engage in discussions about politics in the same space, through commenting and having other kinds of online and offline conversations with others about the stories they see circulating online. This hypothesis is based on what we know about exposure to political information and political discussion, that generally, regardless of medium, there are positive associations found in various studies which extends as well to information found online. [41, 42]

H5: Those with more friends on their network who are politically active, will have greater political knowledge and more political engagement than those who have few politically active friends on their FB network.

One effect of political knowledge, discussion, and interest is political engagement, according to historical political communication literature that examines processes of media influence and interpersonal discussion about politics. [43, 44] Since those with politically active friends on Facebook will see more political information as hypothesized in H4, they are hypothesized to gain more knowledge and consequently, be more likely to engage politically.

## Method

This study is based on an online survey of Filipinos based in the Philippines. The questionnaire contained items that measure FB use, reliance on it for news, reports of pages that they follow, and general political interest and engagement among others. It was constructed using SurveyMonkey which hosted the survey, data collection, and data recording. The questionnaire was posted online through an FB page post containing the link to the questionnaire, which was then promoted to Filipinos between the ages of 15 and 45. A Filipino version of the questionnaire and post was also promoted. Data gathering was conducted between February 1 and March 31, 2016.

The study is an opt-in online survey with informed consent. Participants were informed that their participation is completely voluntary, anonymous, and that they can abandon the survey at any time even after it has been started. The University of the Philippines does not have an Institutional Review Board, but the grant review process includes opportunity for the granting body and the reviewers to reject the proposal based on possible harm to subjects. A signed copy of the Waiver Certification for Ethics Review by the Vice Chancellor of Research and Development of the University of the Philippines, whose office funded this research, is submitted as a supporting document.

A total of 1,555 respondents started the survey; 1,439 answered in English while 116 answered in Filipino. Respondents who fell outside of the age range, those not residing in the Philippines, and those who abandoned the questionnaire before getting to the last question, were screened out. The final sample size is 978, where 41% are male. The average age of the sample is 23 years and the biggest number of them completed high school (44.9%) followed by those who completed college (33.2%). A big majority of the respondents are from Manila (74.2%) and the rest are from other parts of the country.

Owing to the non-probability nature of the sample drawn for this study, this paper makes no claims of representativeness to a broader population in terms of prevalence of social media use or any other descriptive factor. The value of the following results is in the significant multivariate relationships tested, which still hold internal validity even when external validity is weak. [45] The primary objective of this analysis is not to make population inferences, but to test relationships between variables, for which, nonprobability sampling can provide data with value particularly with exploratory research such as this one. The inability of a sample to represent a broader population “does not necessarily negate their usefulness for research”[46].

### Measures

#### FB news reliance

Respondents were asked “If you were not able to see any news about politics and government from FB, how informed would you be about current events?” The response options were Not Informed at All, Only Slightly Informed, Well-Informed, and Fully Informed.

#### Knowledge of politics

Self-reported political knowledge is calculated using two questions. The first is how much do respondents think they know about politics and the second asks whether they typically turn to others for information or others turn to them for information.

#### Interest in politics

Respondents answered one question on political interest on a 4-point scale from Not at all interested in politics to Very interested in politics. The question was “Please tell us how interested you are in national politics.”

#### Political engagement

Offline engagement is a standard measure of the sum of activities respondents said they have done in the past year, including volunteering for a political organization, attending a rally, encouraging another person to vote, being an active member of a group that tries to infleunce public policy or government, volunteering or working for a school-based political party, and displaying a campaign button or sticker.

#### Exposure to FB news

Respondents reported how many of each type of FB page they follow on their FB feed, these include: government offices and agencies, political organizations, politicians and candidates (local and national), news organizations and reporters, political commentators, issue-based groups and other political parties or non-government organizations. For each category, the response options are 0–1, 2–4, and 5 or more. The first three categories were non-news sites; the mean was computed for these, then another mean was computed for the news sites. The two means were summed for a variable that ranged from 2–6 (alpha = 0.85, M = 3.25, SD = 0.99).

#### FB Informs on politics

On a 4-point scale ranging from A Lot to Not at All, respondents were asked if FB helps them with the following: staying informed about current events, staying informed about the local community, learning about political issues that affect them personally, learning about political issues that affect the country, and learning their friends’ political beliefs. The scale is reliable (alpha = 0.84).

#### Discussing politics

Discussing politics is a mean index of three items, one that asks how often they discuss politics with others (4-point scale from Nearly Everyday to Less Often than a Few Times a Month), whether they lead the conversation or listen (0/1), and how much they enjoy talking about politics with friends and family (4-point scale from Very Much to Not at all). The final index ranges from 0.75 to 2.

#### Politically active friends on FB

The mean of five items was computed to represent how many of a respondent’s friends on their FB network are politically interested and active (alpha = 0.88). These include a question on the proportion of their FB friends who are interested in news, how many are politically active, how many frequently post or share news stories, and how many post their personal opinions about politics.

#### Controls

Multivariate regressions run to test the hypotheses posed control for age, educational attainment, sex, income group, interest in politics, attention paid to politics on FB, and how much they follow traditional news (i.e. newspaper, television, radio).

## Results

In the sample, reliance on FB for news was relatively high, with 63% reporting that if they did not obtain news from the platform they would only be slightly informed, and another 4% saying they would not be informed at all. Only 33% say that without FB as a source, they would be well informed or fully informed. The average level of self-reported political knowledge is 2.56 on a 4-point scale with higher values indicating greater knowledge (Standard deviation of the Mean = 0.66). Twenty-eight percent (28%) say that they are usually the source of information by others, and the rest (72%) say that they typically turn to others for political information. Level of political interest in the sample was an average of 2.77 on a self-reported scale from 0 to 4 with higher values indicating higher interest levels (Standard deviation = 0.64). Political engagement offline was measured by asking respondents of a list of 11 possible forms of engagement, how many they engaged in over the past year, the possible range of the resulting index is 0–11 and the overall mean is 3.46 with a standard deviation of 2.44.

On using FB to stay informed about politics, the respondents on average indicated they use FB to stay informed at 2.26 (standard deviation = 0.6) on a scale ranging from 0–4 with higher values indicating greater use of FB for political information. The computed scale for discussing politics ranges from 0.75 to 2.75 with higher values indicating more discussion, the mean is 1.76 and standard deviation 0.5. The index computed for proportion of politically active friends on FB ranges from 0.2–3.8 with higher values indicating more politically interested and active friends (M = 2.4, SD = 0.7).

Bivariate-level tests of hypotheses are reported using Pearson’s R correlation coefficients, while multivariate tests are conducted through Ordinary Least Squares (OLS) regressions which allows for the application of statistical controls to test robustness of bivariate associations. Table 1 reports the Pearson’s r correlations matrix for all variables in hypotheses.

**Table 1:** Pearson’s r correlation coefficients.

	(1)	(2)	(3)	(4)	(5)	(6)	(7)	(8)	(9)
(1) Reliance on FB	1								
(2) Perceived knowledge	.444***	1							
(3) Following news sites and govt agencies on FB	.291**	.389**	1						
(4) Interest in politics	.376**	.415**	.464**	1					
(5) Offline participation (Engagement)	.219**	.327**	.483**	.433**	1				
(6) Traditional news use	.394**	.361**	.381**	.411**	.251**	1			
(7) Having politically active friends on FB	.043	.272**	.285**	.270**	.226**	.191**	1		
(8) FB is source of information	.062	.312**	.305**	.353**	.212**	.218**	.352**	1	
(9) Discussing politics with others	.396*	.532**	.455**	.551**	.434**	.361**	.272**	.312**	1

*p < .05

**p < .01

***p < .0001

Hypothesis 1 posits that those on social media who have lower reliance on FB as a news source would exhibit higher levels of perceived knowledge about politics. Results indicate that at the bivariate level, self-assessed knowledge of politics is moderately and positively correlated with FB news reliance (Pearson’s r = .444*; n = 966). The mean level of knowledge among those who say they are fully reliant on FB for news is 4.3, while for those who would remain well-informed even without FB as a news source is 5.69, and for those who would remain fully informed even without FB it is 6.85. Those on FB who rely less on it as a news source exhibit higher levels of perceived knowledge about politics than those who rely more on it for news. Thus, at the bivariate level, those who are less reliant on FB for news perceive themselves to be more knowledgeable about politics than those who are reliant, supporting hypothesis 1.

To examine the robustness of the association, it was tested in an ordinary least squares regression where knowledge is the main dependent variable and reliance is the main independent variable, other variables were added as controls or as part of the hypothesized effects (Table 2). OLS Regression was run predicting self-assessed political knowledge (Table 2) with news reliance, FB use measures, news exposure measures, and other relevant variables and controls. Each of the three models adds new variables as controls. Model 1 controls for FB-related variables, Model 2 adds controls for following traditional news, interest in poltiics, and discussing politics, while Model 3 adds controls for all demographic variables. Adding each set of controls in different models provides additional information in terms of which controls were likely reducing the predictive value of the main independent variable.

**Table 2:** OLS Regression coefficients predicting self-assessed political knowledge.

	Model 1		Model 2		Model 3	
	B	Beta	B	Beta	B	Beta
	2.487		2.114		1.772	
FB News Reliance	0.601	0.28*	0.469	0.218*	0.386	0.179*
Heavy FB user	0.148	0.076*	0.127	0.065*	0.083	0.043
Uses FB Often	-0.007	-0.091*	-0.005	-0.071*	-0.004	-0.056
Follows news sites and gov’t agencies on FB	0.244	0.189*	0.174	0.135*	0.21	0.163*
Political engagement on FB	0.075	0.026	-0.013	-0.005	-0.027	-0.009
Attention to politics on FB	0.117	0.126*	0.012	0.013	0.01	0.011
FB Informs on politics	0.063	0.03	0.014	0.007	0.011	0.005
FB friends with different views	0.103	0.047	0.1	0.046	0.073	0.034
FB friends politically active	-0.011	-0.006	-0.046	-0.025	-0.027	-0.015
Follows traditional news			0.043	0.024	-0.026	-0.014
Interest in politics			0.042	0.02	0.026	0.013
Discusses politics			0.76	0.302*	0.795	0.315*
Age					0.031	0.159*
Education					0.014	0.014
Female					-0.274	-0.106*
Income group					0.059	0.04
R^2^	0.24		0.298		0.33	
Adjusted R^2^	0.232		0.298		0.318	

*p < .05

**p < .01

***p < .0001

OLS regressions generate two coefficients, Model “Bs” and model betas, the latter is a standardized version of the former. Model “Bs” are changes in the independent variable resulting from changes in the dependent variable, expressed in the unit of the dependent variable. That is, for each unit increase in FB news reliance, there is a 0.61 unit increase in perceived political knowledge, controlling for all other variables in the model. Model betas are standardized partial correlation coefficients between the independent variable and the dependent variable, controlling for the other variables in the model.

With all controls applied, there is a remaining significant association between reliance on FB for news and self-assessed political knowledge (beta = 0.179*). In the same model, the strongest associate of knowledge is discussing politics (beta = 0.315*) followed by following news and government agency pages on FB (beta = 0.163*). Among demographic controls, males and younger respondents are less likely to be politically knowledgeable. The total model explains 33% of the variance of self-assessed political knowledge.

Hypothesis 2 posits that following political officials or institutions on social media will be associated with higher levels of political interest and engagement. Respondents that report following more FB pages of government agencies and news websites are more likely to say they are interested in politics (Pearson’s r = 0.464; n = 925), more likely to engage in offline political activities (Pearson’s r = 0.3434; n = 823), and more likely to display online political engagement (r = 0.483; n = 886). Controlling for following news on traditional channels and other variables in the model, hypothesis 2 is supported.

With controls for all the same variables as in Table 2, the relationship between following news pages online and political engagement remains significant such that those who report following government agency and news sites on FB tend to be more politically engaged offline (b = 0.267*). The full model results can be found in Table 3, wherein the column for Model 4 coefficients reflects results controlling for demographic variables.

**Table 3:** OLS Regression coefficients predicting political engagement offline.

	Model 1		Model 2		Model 3		Model 4	
	B	Beta	B	Beta	B	Beta	B	Beta
	-1.8		-2.917		-3.124		-3.83	
FB News Reliance	0.305	0.076*	0.103	0.026	0.052	0.013	-0.018	-0.005
Heavy FB user	0.176	0.046	0.135	0.035	0.118	0.031	0.086	0.023
Uses FB Often	0.009	0.059	0.011	0.076*	0.011	0.078*	0.012	0.083*
Follows news sites and gov’t agencies on FB	0.761	0.308*	0.643	0.26*	0.625	0.253*	0.66	0.267*
Political engagement on FB	0.235	0.043	0.207	0.038	0.206	0.037	0.239	0.043
Attention to politics on FB	0.199	0.109*	0.022	0.012	0.021	0.012	0.017	0.009
FB Informs on politics	0.073	0.018	-0.023	-0.006	-0.024	-0.006	-0.058	-0.014
FB friends with different views	-0.003	-0.001	-0.013	-0.003	-0.029	-0.007	-0.052	-0.012
FB friends politically active	0.099	0.027	0.079	0.022	0.088	0.024	0.065	0.018
Follows traditional news			-0.078	-0.022	-0.083	-0.024	-0.115	-0.033
Interest in politics			0.654	0.159*	0.648	0.157*	0.626	0.152*
Discussing politics			0.663	0.134*	0.584	0.118*	0.635	0.128*
Political knowledge					0.105	0.055	0.08	0.042
Age							0.017	0.047
Education							0.092	0.051
Female							0.06	0.012
Income group							0.014	0.005
R^2^	0.228		0.261		0.263		0.27	
Adjusted R^2^	0.219		0.25		0.251		0.255	

*p < .05

**p < .01

***p < .0001

Hypothesis 3 is likewise supported such that controlling for traditional news use, those with more politically active friends on FB have higher levels of exposure to political content online, as measured by whether FB informs them about politics (Pearson’s r = 0.352; n = 947).

The same holds true at the bivariate level with Hypothesis 4, whereby there is a positive correlation between FB being a source of information about politics, and discussing politics more often with others (r = 0.312, n = 947). Table 4 shows regression results for models predicting political discussion. It is evident that with multiple controls, there is no longer a significant association between FB being a source of information about politics, and political discussion. Significant predictors of discussing politics with others are non-reliance on FB for news, paying attention to politics on FB, being interested in politics, and following news site pages on the social media platform.

**Table 4:** OLS Regression coefficients predicting political discussion.

	Model 1		Model 2		Model 3	
	B	Beta	B	Beta	B	Beta
(Constant)	0.406		0.189		0.23	
FB News Reliance	0.144	0.169*	0.097	0.114*	0.104	0.122*
Heavy FB user	0.024	0.031	0.019	0.024	0.023	0.03
Uses FB Often	-0.002	-0.055	-0.001	-0.037	-0.001	-0.04
Follows news sites and govt agencies on FB	0.079	0.154*	0.05	0.097*	0.043	0.085*
Political engagement on FB	0.106	0.092*	0.11	0.095*	0.11	0.095*
Attention to politics on FB	0.128	0.348*	0.098	0.266*	0.098	0.267*
FB Informs on politics	0.05	0.061	0.022	0.027	0.023	0.028
FB friends with different views	0.003	0.004	0	0.001	0.002	0.002
FB friends politically active	0.042	0.057	0.035	0.048	0.031	0.043
Follows traditional news			0.024	0.033	0.033	0.045
Interest in politics			0.191	0.233*	0.191	0.234*
Age					-0.005	-0.071*
Education					0.013	0.035
Female					0.014	0.014
Income group					-0.006	-0.01
R^2^	0.394		0.428		0.431	
Adjusted R^2^	0.387		0.421		0.421	

*p < .05

**p < .01

***p < .0001

Hypothesis 5 posits that those with more politically active friends on their network will have greater political knowledge and more political engagement than those who have few politically active friends on their FB network. Tables 2 and 4 show that this hypothesis is not supported. There is no significant relationship between having a larger number of politically active friends on FB and being more politically knowledgeable and engaged.

## Discussion

In this research we seek to examine how “reliance on FB” for political news influences citizens’ political knowledge and participation. We understand reliance to mean that if one does not receive news stories through the FB platform, there would be no alternative source and the result is low knowledge of politics. There was a special interest in capturing these associations among relatively young people, those below the age of 45 years old.

The online survey of young Filipinos on FB result in support for most hypotheses posed in this study. Respondents who say they are fully reliant on the platform for news, think of themselves as less politically knowledgeable than those who say they are not reliant. This association holds even while controlling for news consumption in traditional news channels. Following the FB pages of political officials or institutions on social media is associated with higher levels of interest and engagement among respondents. Moreover, respondents who report having more friends who are politically active report higher levels of exposure to political content online.

The hypothesis that those who see themselves as sources of political information during conversations are also more likely to discuss politics with others was not supported by the data. Also not supported was the hypothesis that those with more friends on their network who are politically active will have higher levels of knowledge and engagement than those who have fewer politically active friends on FB.

FB’s growth as a significant player in the news market does not happen in a vacuum without the convergence of certain factors. It happens within the context of a continued existence of traditional news, news consumer’s social networks, and an effort among news sources (e.g. government agencies) to engage directly with their constituents through the platform. This research attempts to capture some portion of this dynamic.

While reliance on FB for news is positively associated with self-assessed political knowledge, it is not a significant predictor of engagement. People do receive information from FB, and for some of them it is their only source of news. Without FB, it is possible that these individuals would have no other source of news and thus, would have lower levels of political knowledge. In that sense, FB is bringing in new audiences for public affairs, through more incidental forms of exposure in FB feeds than through information-seeking behaviors such as checking news websites. Those who follow official government pages or official news pages on FB are more knowledgeable, even when controlling for reliance and other measures in this study. Thus, the remaining significant effect of reliance on knowledge captures incidental exposure, the kind that is not a result of stories appearing across one’s news feeds after having followed a news outlet’s FB page. If this interpretation is correct, then it is theoretically consistent with the lack of influence of FB reliance on political engagement. The reliant are those who have little interest to begin with, they see stories on their feed only because their social network share them (in the extreme scenario), and thus, they are least likely to have interest in political action.

The lack of support for hypothesized associations between political activity of friends on FB and political knowledge and engagement is curious. Momentarily setting aside the possibility that the reason is methodological, the apparent lack of significant relationship is intriguing. If you have many friends on your feed who are politically active and interested, it stands to reason that either through persuasion or exposure or both, you would be more willing to engage in political action. The absence of a correlation, where the significant correlates to offline political engagement are interest and following news, suggests that FB may not be an effective way to mobilize those whose current interest in politics is either null or weak.

There are a number of limitations to this study, foremost of which is its reliance on self-reports on knowledge and reliance on FB for news. Studies that have examined different measures of political knowledge [47] conclude that “knowledge tests” are the best most objective indicators, and self-reported ones are questionable in their ability to capture real knowledge. That said, the results reported in Table 1 suggests that the measure captures a substantial amount of variance, such that it correlates with variables that it is usually associated with (e.g. discussing politics, age, sex). In other words, while not the ideal measure, it captures political knowledge in some way. Follow-on research could focus on greater methodological work in measuring “reliance” using a more expansive battery of questions that capture all other potential sources of political information.

Since the study’s respondents are only Filipino audiences, the degree to which findings are applicable to outside markets remains an open question. However, we submit that the reliance on Facebook for news delivery and the influence it has had on the nature of the news consuming public is felt in many countries in the world. Replications of the same hypothesis tests, using the same questionnaire, in different markets are welcome and would provide a nuanced cross-country picture of the scope and breadth of the impact that FB has in individual countries.

This research presents possible take-off points, especially as FB itself makes stronger its claim of being the main portal through which citizens learn about public affairs, discuss it, organize action around it, and define themselves as citizens. Various lines of inquiry on the implications of FB on news production and consumption remain open, with the former commanding greater attention and faster development in the literature. The evolution of news consumption through FB and its effect on the political opinions and behaviors of citizens varies across countries of differing political regimes, levels of development, and access to technology.

In a country like the Philippines where the population does not have a strong history of journalism and news use, FB’s role in the shaping of politics and citizenship is likely going to be bigger, and will happen faster than in more developed and stable democracies. The potential of FB to bring in and create larger audiences for news is strong, but this has also meant that the bar for what counts as “news” is low for most of these new audiences. It is an evolution of citizenship and political engagement that will be influenced disproportionately by FB, what it decides to do, and how it decides to define its role in the news media landscape.

## Supporting information

S1 File Survey questionnaire. (PDF)

S2 File Survey dataset. (CSV)
