# Supplementary material for: Reliance on Facebook for news and its influence on political engagement
Source: PLoS One. 2019 Mar 19;14(3):e0212263. doi: 10.1371/journal.pone.0212263 (PMC6424427; doi:10.1371/journal.pone.0212263)
Supplement: S1 File — (PDF) [file pone.0212263.s001.pdf]

Welcome to our survey on politics and social media

Thank you for participating in this survey about social media and your interest in social issues, politics, and government. It will only take you about 15-20 minutes to complete this survey. Kindly answer all questions as honestly as possible as there are no right or wrong answers.

Your responses will be kept strictly confidential and we will not be asking any questions that will allow us to identify you. Your participation is voluntary and you are free to stop filling out the survey, at any point, if you wish. You will not be named in any report arising from this survey.

This survey is being conducted for academic purposes by faculty of the University of the Philippines College of Mass Communication and is supported by the University. It is not supported by or connected to any electoral campaign or candidate nor any news organization.

If you have any questions or concerns, you may get in touch with the Project Team through Clarissa C. David at ccdavid2@up.edu.ph.

Background Information

Are you male or female?

- ◯ Male
- ◯ Female

Where do you currently live?

- ◯ Metro Manila
- ◯ Luzon outside of Metro Manila
- ◯ Visayas
- ◯ Mindanao
- ◯ Other (please specify)

[ ]

What is the highest level of school you have completed or the highest degree you have received?

- ◯ Some elementary
- ◯ Completed elementary
- ◯ Some high school
- ◯ Completed high school
- ◯ Vocational or technical degree
- ◯ Some college but no degree
- ◯ Completed college

In what year were you born? (enter 4-digit birth year; for example, 1976)

[ ]

Which of the following electronic devices do you use? (Please select all that apply.)

- [ ] Desktop Computer
- [ ] Laptop Computer
- [ ] Tablet computer (e.g. iPad, Samsung Galaxy)
- [ ] Smart phone or mobile phone that can connect to the Internet
- [ ] I don't use any electronic device

Below is an income scale on which 1 indicates the lowest income group and 6 the highest income group in the Philippines. We would like to know in what group your household is. Please pick the appropriate number.

Please tell us if you are currently a student or working.

- ( ) Student
- ( ) Working
- ( ) Not a student and not working
- ( ) Other (please specify)

Aside from the election, please tell us up to three of the most important problems the country is facing today.

Most important problem	
Second most important problem	
Third most important problem	

Concerning the Presidential election, what do you think are the three most important problems that the candidates are facing today?

Most important	
Second most important	
Third most important	

Interest in politics

Generally speaking, how much do you know about politics and government?

- ◯ Very much
- ◯ Much
- ◯ Little
- ◯ Very little

Do you typically turn to other people for information about government and politics, or do they typically turn to you?

- ◯ I turn to others for information
- ◯ Others turn to me for information

Are you a registered voter?

- ◯ Yes
- ◯ No
- ◯ Unsure / Don’t know

Discussions about politics

How often do you discuss government and politics with others?

- ◯ Nearly everyday
- ◯ A few times a week
- ◯ A few times a month
- ◯ Less often

When you talk to your friends and family about government and politics, do you listen to the conversation more than lead, or lead the conversation more than listen?

- ◯ I listen to the conversation more than lead
- ◯ I lead the conversation more than listen

How much do you enjoy talking about government and politics with friends and family?

- ◯ Very much
- ◯ Much
- ◯ Not much
- ◯ Not at all

Opinions about government

Below are statements about politics and government that some people believe and others do not. Please tell us if you agree or disagree with each of them.

	Strongly disagree	Disagree	Neither disagree nor agree	Agree	Strongly agree
People like me don't have any say in what the government does	◯	◯	◯	◯	◯
The nation is run by a powerful few, ordinary citizens cannot do anything about it	◯	◯	◯	◯	◯
The government is really run for the benefit of all people	◯	◯	◯	◯	◯
When something is run by government, it is usually inefficient or wasteful	◯	◯	◯	◯	◯
We can trust what politicians say	◯	◯	◯	◯	◯
Politicians pay more attention to taking sides rather than to planning or carrying out policy	◯	◯	◯	◯	◯
Politicians are out of touch with life in the real world	◯	◯	◯	◯	◯
Politicians put their own interests ahead of the public's interest	◯	◯	◯	◯	◯

Political activities

Please tell us if you have done any of the activities listed below in the past year. (Please select all that apply.)

- [ ] Been an active member in a group or organization for a political or social cause
- [ ] Volunteered or worked for a political or electoral organization
- [ ] Participated in any fundraising activities for/through your school or community
- [ ] Displayed campaign buttons, signs or stickers
- [ ] Signed a written petition
- [ ] Attended a political rally, speech, or organized protest of any kind
- [ ] Attended a political meeting on local or national affairs
- [ ] Been an active member of a group that tries to influence public policy or government
- [ ] Worked with fellow citizens to solve a problem in your community
- [ ] Encouraged another person to vote
- [ ] Volunteered or worked for a school-based political party

Interest in politics

Please tell us how interested you are in the following

	Not at all interested	Somewhat interested	Interested	Very interested
Following national politics	◯	◯	◯	◯
Following local politics	◯	◯	◯	◯
Following news about elections	◯	◯	◯	◯
Following news about specific social issues (for example, the environment, education, health)	◯	◯	◯	◯
Following international news	◯	◯	◯	◯

Following news

How often do you read or watch political news from each of these in a typical week?

	Less than once a week	1-2 days a week	3-4 days a week	Everyday
Television	○	○	○	○
Radio	○	○	○	○
Newspapers and news magazines	○	○	○	○

How often do you read or watch political news on the Internet? (including social media)

- ○ Never
- ○ Not often
- ○ 1-2 days a week
- ○ 3-4 days a week
- ○ Everyday

Use of social media

Which of the following social networking websites do you regularly use? (Check all that apply)

- [ ] Google+
- [ ] Twitter
- [ ] LinkedIn
- [ ] Instagram
- [ ] I do not regularly use any social networking websites

Would you say you are a heavy, medium, or light user of the social networking site Facebook?

- ○ Heavy user
- ○ Medium user
- ○ Light user
- ○ I do not use Facebook

Facebook use

Which of these do you use most often to access your Facebook account?

- ◯ Desktop Computer
- ◯ Laptop Computer
- ◯ Tablet computer (e.g. iPad, Samsung Galaxy)
- ◯ Smart phone or mobile phone that can connect to the Internet
- ◯ I do not access Facebook

How often do you read your Facebook feed?

- ◯ Several times a day
- ◯ About once a day
- ◯ A few times a week
- ◯ Every few weeks
- ◯ Less often

How much time in a typical day do you spend reading and checking your Facebook feed, include the time you spend reading articles and watching videos that you find through Facebook?

- ◯ Less than 30 minutes a day
- ◯ 1-2 hours a day
- ◯ 3-4 hours a day
- ◯ More than 4 hours a day

News on Facebook

You said that you read or watch some of your news on the Internet, please tell us which of the following kinds of sources you usually use to get your political news online. You may check as many as applicable.

- ☐ Facebook feed
- ☐ Twitter feed
- ☐ Philippine news websites (for example, Inquirer.net, Rappler.com, ABS-CBNnews.com)
- ☐ Foreign news websites (for example, CNN.com, theguardian.com, newyorktimes.com)
- ☐ Political blogs

How much attention do you pay to posts about government and politics on Facebook?

- ○ A lot
- ○ Some
- ○ Not much
- ○ Not at all

If you were not able to see any news about politics and government from Facebook, how informed would you be about current events?

- ○ Not informed at all
- ○ Only slightly informed
- ○ Well-informed
- ○ Fully informed

Following news sources on Facebook

Do you “like” or “follow” any of the following sources of news on Facebook? If so, roughly how many?

	0-1	2-4	5 or more
News organizations and reporters (example: ABS-CBN News, Rappler, Jessica Soho)	○	○	○
Political commentators (example: Raissa Robles, Juana Change)	○	○	○
Issue-based groups other than political parties such as NGOs (example: Greenpeace, PETA, Filipino Freethinkers)	○	○	○

Engagement with news on Facebook

When you see a political news story on your FB feed, how often do you click on the link to read the whole story or watch the video?

- Never
- Rarely
- Some of the time
- Most of the time
- Always

In a typical week, how many times do you do each of the following on your Facebook account?

	0-2 times	3-6 times	7 or more times
Comment on a news story or blog you read online			
"Like" a news story or blog you read online			
Post/repost a link to a news story or blog			
Write your own opinion or comment about political news on your FB status			

Learning about politics on Facebook

Please tell us how much Facebook helps you with the following

	A lot	Some	Not much	Not at all
Staying informed about current events and public affairs	○	○	○	○
Staying informed about the local community	○	○	○	○
Learning about political issues that affect me personally	○	○	○	○
Learning about political issues that affect the country	○	○	○	○
Learning my friends' political beliefs	○	○	○	○

Facebook Friends' Political Interest

Some people are interested in news about government and politics, while others are not. How much are your Facebook friends interested in news about government and politics?

- Most of my FB friends are interested in news
- About half are interested
- Less than half are interested
- Very few are interested
- None of my FB friends are interested in news

How many of your Facebook friends have political views different from your own?

- Most
- Some
- Very few
- None

Among your FB friends, how many would you say are politically active?

- Most of my FB friends are politically active
- About half are politically active
- Less than half are politically active
- Very few are politically active
- None of my FB friends are politically active

How many of your friends on FB frequently "post" or "share" news stories about politics and government on their feed?

- Most of my FB friends
- About half
- Less than half
- Very few
- None

How many of your friends on FB frequently post their personal opinions about political and government issues?

- ◯ Most of my FB friends
- ◯ About half
- ◯ Less than half
- ◯ Very few
- ◯ None

Online political engagement

Which of the following online activities relating to government and the elections have you done in the last 3 months?

- ☐ Look for information online about candidates' positions on issues
- ☐ Encouraged others to take action on a social or political issue that is important to you
- ☐ Contacted a government official by Facebook about an issue that is important to you
- ☐ Watched video online about the candidates or the election
- ☐ Sent messages by email, Facebook, or Twitter related to the campaign or the elections to others
- ☐ Used the Internet to organize or get information about in-person meetings to discuss political issues
- ☐ Revealed online which candidate you are voting for
- ☐ Used the Internet to to research or "fact check" claims made during the campaign

Some people do not follow political or government pages on their Facebook accounts, some people do. How many of each type do you follow on your FB?

	0-1	2-4	5 or more
Government offices or agencies (for example, Department of Health, municipal government, provincial government)	○	○	○
Political organizations (for example, political parties, NGOs, activist groups, student councils)	○	○	○
National politicians and candidates	○	○	○
Local politicians and candidates	○	○	○

End of Survey

Thank you for participating in this survey. Your answers will be kept confidential and any reports or papers published from this data will not contain any identifying information. If you have any concerns or questions please leave them in the comment box below or email the study leader at ccdavid2@up.edu.ph.

If you would like to receive a copy of the paper once it is completed please leave your email address below.
